# USP37 Promotes Lung Cancer Cell Migration by Stabilizing Snail Protein *via* Deubiquitination

**DOI:** 10.3389/fgene.2019.01324

**Published:** 2020-01-10

**Authors:** Jiali Cai, Mengying Li, Xiang Wang, Lei Li, Qi Li, Zhaoyuan Hou, Hao Jia, Shiyuan Liu

**Affiliations:** ^1^ Department of Radiology, Changzheng Hospital, Second Military Medical University, Shanghai, China; ^2^ Hongqiao Institute of Medicine, Shanghai Tongren Hospital/Faculty of Basic Medicine, Shanghai Jiaotong University School of Medicine, Shanghai, China; ^3^ Department of Thoracic Surgery, Lanling People's Hospital, Lanling County, Linyi, China; ^4^ Shanghai Key Laboratory for Tumor Microenvironment and Inflammation, Department of Biochemistry & Molecular Cellular Biology, Shanghai Jiaotong University School of Medicine, Shanghai, China

**Keywords:** deubiquitination, USP37, Snail, cell migration, lung cancer

## Abstract

Snail is a prominent epithelial–mesenchymal transition (EMT) transcription factor and promotes metastasis. However, Snail protein is unstable and is quickly degraded through ubiquitination-mediated proteasome pathway. Deubiquitinases prevent Snail degradation by regulating the ubiquitination-mediated hydrolysis process. Our studies demonstrate that a deubiquitinating enzyme (DUB) family member, USP37, can deubiquitinate Snail and prevent degradation of Snail. USP37 is co-localized with Snail in the nucleus. Biologically, upregulated expression of USP37 promotes lung cancer cell migration, while depletion of Snail abolishes the effect of USP37. These data demonstrate that USP37 is a Snail-specific deubiquitinase and also indicate a potential therapeutic target for metastasis.

## Introduction

Snail is a member of SNAG domain-containing zinc finger transcription factors and a major regulator of epithelial–mesenchymal transition (EMT) and metastasis in various tumor types (Wang et al., 2013; Diaz et al., 2014). Snail can directly bind to the E-boxes to repress expression of a large pool of genes that controls epithelial identity and convert normal epithelial cells into mesenchymal cell phenotype (Yu et al., 2017). Mechanistically, Snail recruits multiple repressive protein complexes involved in histone deacetylation, methylation, and ubiquitination to its target promoters and exerts its repressive function (Vree et al., 1987; Spencer et al., 1999; Peinado et al., 2004; Zhou et al., 2004; Dong et al., 2013). Clinically, Snail expression is associated with resistance to chemotherapy, decreased survival, high recurrence rates, and poor prognoses (Suresh et al., 2016). In accordance with its profound role in development and diseases, the level of Snail protein is tightly regulated through various extracellular signaling including transcription or protein degradation (Park et al., 2010; Suresh et al., 2016; Gudey et al., 2017).

Snail is a labile protein with an estimated half-life of about 30 min (Park et al., 2010; Wang et al., 2015). Several studies demonstrated that Snail can be quickly degraded *via* ubiquitination-mediated protein hydrolysis pathway (Suresh et al., 2016; Gudey et al., 2017). Several ring domain-containing E3 ligases, such as Fbxl5 (Wu et al., 2015), Fbxl14 (Vinas-Castells et al., 2010; Diaz and de Herreros, 2016), Fbxw1 (β-Trcp) (Vinas-Castells et al., 2014; Diaz and de Herreros, 2016), Fbxo11 (Zheng et al., 2014; Jin et al., 2015), and SCF-FbxO45 (Xu et al., 2015), have been identified to ubiquitinate Snail and promote its degradation in various cell types. However, ubiquitination can be reversed by deubiquitinating enzymes (DUBs) (Komander, 2010; Mevissen and Komander, 2017). Approximately 100 putative DUBs have been found in human genome (Reyes-Turcu et al., 2009), which can be divided into six subfamilies: ubiquitin-specific proteases (USPs), ovarian tumor proteases (OTUs), ubiquitin-C-terminal hydrolases (UCHs), Josephin, the motif interacting with ubiquitin-containing novel DUBs (MINDYs), and JAB1/MPN/MOV34 metalloproteases (JAMMs) (Hochstrasser, 1995). DUBs may directly interact with substrates or indirectly bind to an adaptor protein such as an E3 ubiquitin ligase to remove ubiquitin from the targeted proteins (Mevissen and Komander, 2017). The balance between ubiquitination and deubiquitination is essential for maintaining essential biological processes in the cells. DUB3 (Liu et al., 2017; Wu et al., 2017), OTUB1 (Zhou et al., 2018), and USP27X (Lambies et al., 2019) have been identified as specific DUBs to stabilize Snail and play important roles in Snail-mediated tumor metastasis. In this study, we discover that USP37 directly binds Snail and markedly improves Snail protein stability through its deubiquitinase activity. The increased stability of Snail protein eventually promotes lung cancer cell migration and enhances cancer metastasis.

## Materials and Methods

### Plasmids, Antibodies, and Reagents

The human USP37 cDNA was subcloned into pcDNA3.1-Flag vector or PCDH vector and USP37-C350S was made using PCR-based site-directed mutagenesis method. All other constructs were generated using standard molecular cloning methods and were confirmed by DNA sequencing. Antibodies were commercially purchased: anti-USP37 (rabbit, 18465-1-AP, Proteintech), anti-Snail (rabbit, 3879, Cell Signaling), anti-Ubi (mouse, sc-8017, Santa Cruz), anti-actin (mouse, 60008-1-lg, Proteintech), anti-N-cadherin (mouse, 33-3900, Invitrogen), anti-HA (mouse, MMS-101P, Covance), anti-Flag (mouse, F3165, Sigma), anti-Flag (rabbit, F7425, Sigma), anti-Myc (mouse, 13-2500, Invitrogen), anti-GAPDH (mouse, 60004-1-Ig, Proteintech), normal IgG (rabbit, sc-2027, Santa Cruz), and GSH beads (GE). MG132 and cycloheximide (CHX) were obtained from Sigma.

### Western Blot, Co-IP, and GST Pull-Down

The co-immunoprecipitation (co-IP), Western blotting, and glutathione S-transferase (GST) pull-down assays were described previously (Jia et al., 2017). In brief, cells were lysed 48 h of post-transfection in buffer containing 20 mM/L Tris-HCl (pH 8.0), 150 mM/L NaCl, 2.5 mM/L EDTA, 0.5% NP40, 0.1 mM/L phenylmethylsulfonyl fluoride (PMSF), and protease inhibitor cocktail. Lysates were analyzed by sodium dodecyl sulfate polyacrylamide gel electrophoresis (SDS-PAGE) and Western blot assays. Lysates were pre-incubated with protein A/G PLUS-agarose beads for 1 h and the beads were removed by centrifugation. The resulting supernatants were then incubated with antibodies against the indicated epitope tags followed by incubation with protein A/G PLUS-agarose (SC-2003, Santa Cruz). Beads were washed three times with cell lysis buffer and the co-eluted proteins were analyzed by SDS-PAGE and Western blot assays.

In the GST pull-down assay, GST-Snail and His-Flag-USP37 were expressed in *Escherichia coli* BL21 (DE3) cells, which were induced by isopropyl-β-d-thiogalactoside (IPTG). The GST-tagged Snail protein was purified by Glutathione Sepharose beads (17-0756-01, GE Healthcare) and His-Flag-USP37 was purified with Ni beads (17-5318-06, GE Healthcare).

### Cell Culture and Transfection

Cell lines human lung cancer H1299, H1975, and human embryonic kidney 293T cells were obtained from American Type Culture Collection (ATCC) and maintained in RPMI1640 or in Dulbecco's modified Eagle's medium (DMEM) supplemented with 10% heat-inactivated fetal bovine serum (FBS), 2 mM l-glutamine, and penicillin (50 U/ml)/streptomycin (50 µg/ml) at 37°C in the presence of 5% CO_2_. Transfections were performed using Lipofectamine 3000 (Invitrogen, Carlsbad, CA, USA) according to the manufacturer's instructions.

### Immunofluorescence Assay

Flag-USP37 and HA-Snail plasmids were co-transfected into H1299 cells and 24 h post-transfection. H1299 cells were permeabilized with 0.1% Triton X-100 for 30 min at room temperature, washed with phosphate-buffered saline (PBS), and blocked with PBS containing 5% bovine serum albumin for 1 h at room temperature. Cells were treated with primary antibodies overnight at 4°C. Cells were rinsed with PBS and then incubated with secondary antibody for 1 h at room temperature. The slices were counterstained with diamidino phenylindole (DAPI) and examined using laser scanning confocal microscope (Nikon, Japan). The antibodies used in these assays are as follows: anti-HA (mouse, MMS-101P, Covance), anti-Flag (rabbit, F7425, Sigma), secondary antibodies (488 nm, donkey anti-mouse and 568 nm, donkey anti-rabbit). Images were taken with confocal microscopy.

### shRNA Knockdown and CHX Chase Assay

The short hairpin RNA (shRNA) oligonucleotides were transfected using Lipofectamine 3000 (Invitrogen, Carlsbad, CA, USA). Supernatants containing viruses were packed from 293T cells. When growing to 60–80% confluence, H1299 and H1975 were infected with viral supernatants and 5 μg/ml puromycin was added to select the stable cells. shRNAs against Snail were as follows: shRNA#1 5′-CCACTCAGATGTCAAGAAGTA-3′.

For the CHX chase assay, CHX was added to the cell culture medium at a concentration of 50 μg/ml, and the cells were harvested at the indicated time points (0, 1, 2, 4, 6, 8hrs). The harvested cells were prepared for Western blotting assays with anti-Snail, anti-Flag, and anti-actin antibodies.

### Cell Migration Assays

Cells were harvested after serum-free starvation for 12 h and were resuspended in plain DMEM media. Twenty thousand cells were applied to 8-μm pore Transwell filters (Corning). DMEM containing 10% FBS were added to the bottom chamber as attractants. After incubation for 24 h, the cells on the top side of the filter were removed by washing. The attached cells at the bottom of the filter were fixed with 4% paraformaldehyde and were stained with colloidal staining method. Each group was performed in triplicate. Experiments were independently repeated three times.

### Statistical Analysis

Data shown as mean ± SD were analyzed by independent Student's *t* test.

## Results

### Deubiquitinase USP37 Interacts With Snail

**Figure 1:**
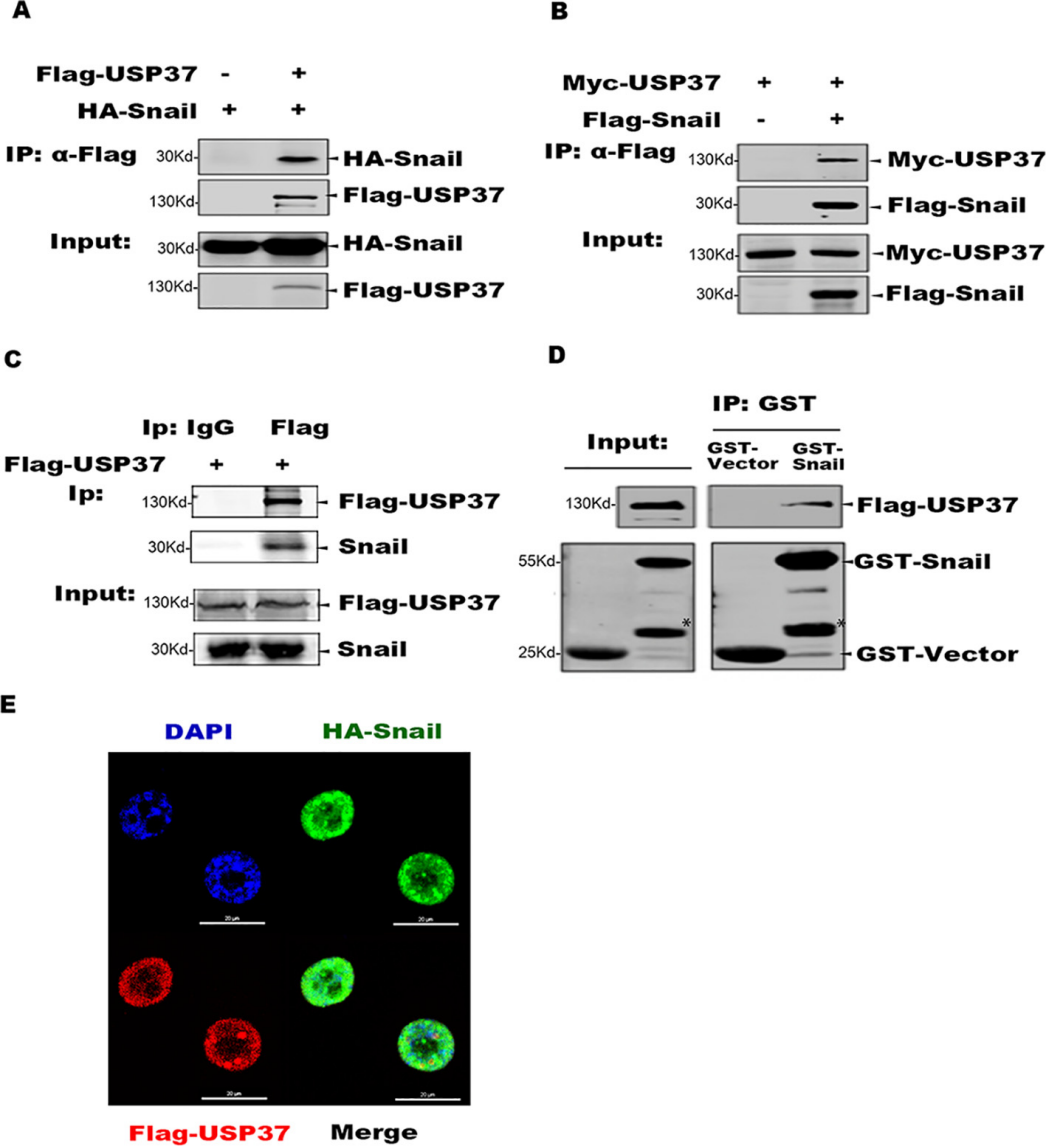
USP37 directly interacts with Snail. **(A)** USP37 can immunoprecipitate Snail. Plasmids encoding Flag-USP37 and HA-Snail were transiently co-expressed in 293T cells and co-immunoprecipitation (co-IP) assays were performed with Flag-M2 beads. The co-eluted proteins were detected by Western blot assays with HA antibody. **(B)** Snail can immunoprecipitate USP37. Plasmids encoding Myc-USP37 and Flag-Snail were transiently co-expressed in 293T cells and co-IP assays were performed with Flag-M2 beads. The co-eluted proteins were detected by Western blot assays with Myc antibody. **(C)** USP37 interacts with endogenous Snail in lung cancer H1299 cells. Flag-USP37 was transiently expressed in H1299 cells and co-IP assays were carried out using Flag antibody. The co-eluted Snail was detected using anti-Snail antibody. **(D)** Glutathione S-transferase (GST) and GST-Snail were expressed and purified in *E. coli* BL21 cells and Flag-USP37 proteins expressed and prepared from 293T, respectively. *In vitro* pull-down assays were performed by using GST beads and the co-eluted USP37 was detected with Flag antibody. *Non-specific band. **(E)** Flag-USP37 and HA-Snail plasmids were co-transfected into H1299 cells, and 24 h post-transfection, immunofluorescence was performed by using primary Flag (rabbit) and HA (mouse) antibodies and secondary antibodies (488 nm, donkey anti-mouse and 568 nm, donkey anti-rabbit). Images were taken with confocal microscopy. *Scale bars*, 20 µm.

To identify specific DUBs which can stabilize Snail, we first transfected plasmids encoding 79 DUBs into 293T cells stabling the expression of Flag-Snail, and the transfected cells were prepared for Western blotting analysis. Besides the recently reported DUB3 (Liu et al., 2017; Wu et al., 2017), we also found USP37 as a potential DUB to stabilize Snail protein (data not shown). We further examined the interaction between Snail and USP37 by performing co-IP assays. Plasmids encoding Flag-USP37 and HA-Snail were co-expressed in 293T cells. Co-IP assays were performed with Flag antibody and the co-eluted proteins were detected with HA antibody. Also, USP37 was immunoprecipitated with Snail (**Figures 1A, B**). Our studies showed that ectopically expressed Flag-USP37 was able to immunoprecipitate endogenous Snail protein in lung cancer H1299 cells (**Figure 1C**).

We also expressed GST-Snail in *E. coli* BL21 cells, which was mixed with Flag-USP37 protein purified from 293T cells, and co-IP assays were carried out with GST beads. The Western blot assays showed that GST-Snail efficiently co-eluted with USP37, indicating a direct interaction between Snail and USP37 (**Figure 1D**). Moreover, we performed indirect immunofluorescence (IF) assay to examine the subcellular localization of Snail and USP37 in 293T and H1299 cells.

HA-Snail and Flag-USP37 were transiently expressed either separated or combined. The following IF assays were performed with a rabbit Flag antibody or mouse HA antibody. Confocal microscopy image showed that both Snail and USP37 were predominately localized in the nucleus with similar distribution patterns (**Figure 1E**).

### USP37 Is Essential for Snail Stabilization

**Figure 2:**
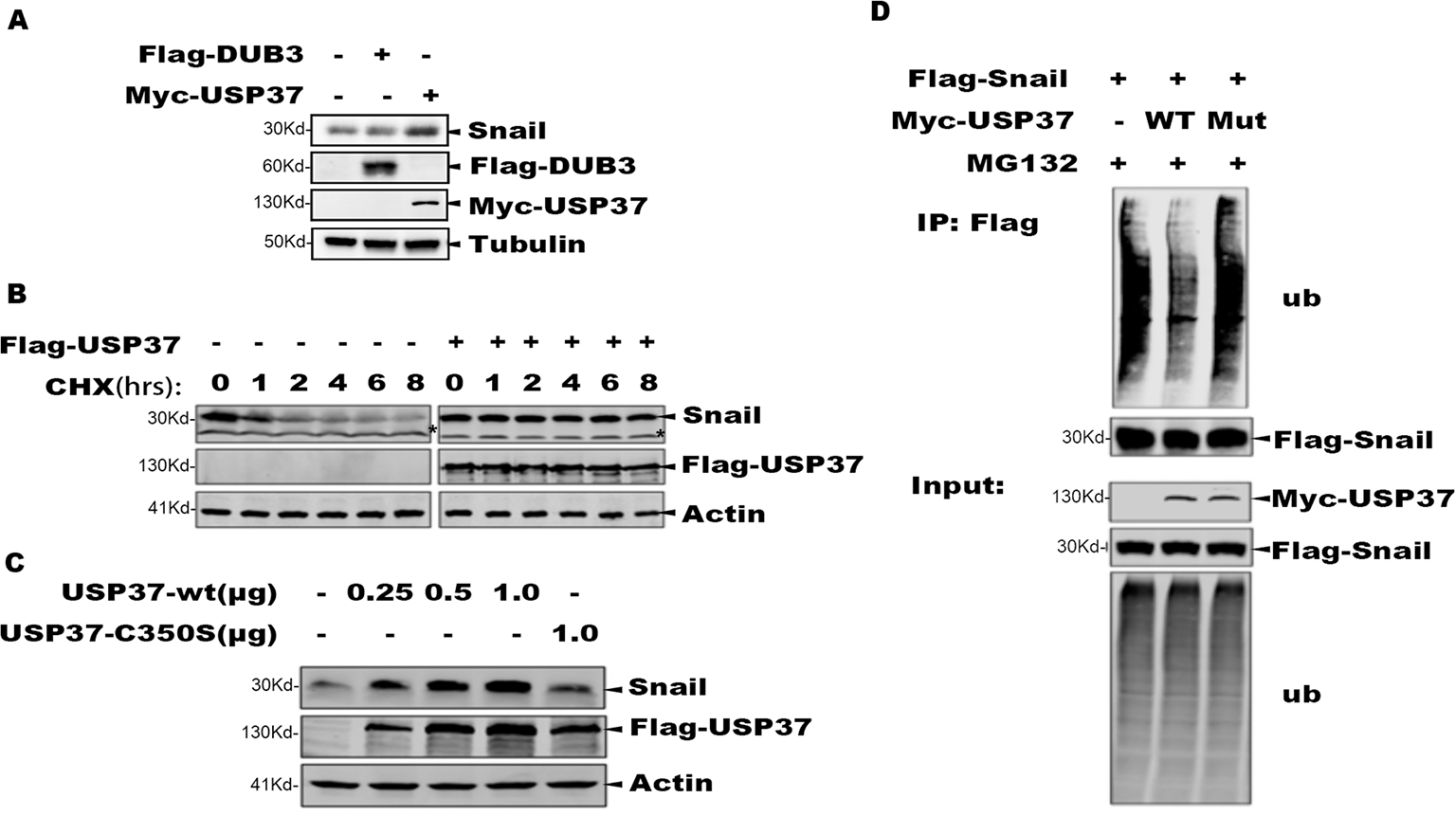
The deubiquitinase activity of USP37 is required for Snail stability. **(A)** Validation of the Dub3 and Usp37 in H1299 cells. Flag-Dub3 and Myc-Usp37 were transfected into H1299 cells, respectively, and endogenous Snail protein levels were measured by Western blot after 48 h. **(B)** USP37 prevents Snail protein degradation. Flag-USP37 or vector control plasmids were transfected into 293T-Flag-Snail cells, and 48 h post-transfection the resulting cells were treated with cycloheximide for various time periods. Whole cell extracts were prepared and Western blot assays were performed using Snail antibody and Flag antibody. Actin level was used as a loading control. *Non-specific band. **(C)** The deubiquitinase activity is required for USP37 to stabilize Snail. 293T-Flag-Snail cells were transfected with plasmids of USP37-WT (0.25, 0.5, and 1.0 µg) and USP39-Mut (1.0 µg). After 48 h, the expression level of Snail was detected by Western blot assays. **(D)** USP37 decreases the ubiquitination level of Snail. Myc-USP37-WT and Myc-USP37-Mut plasmids were transiently expressed in 293T-Flag-Snail cells and the resulting cells were treated with MG132 for 8 h before being harvested. Snail protein was enriched with Flag-M2 beads and ubiquitinated Snail was detected with Ub antibody.

It has been reported that DUB3 can stabilize Snail and play an important role in Snail-mediated tumor metastasis (Wu et al., 2017). We transfected Flag-Dub3 and Myc-Usp37 into H1299 cells, respectively, and endogenous Snail protein levels were measured by Western blot (WB) after 48 h. These data indicate that Usp37 have phenotypes with a more important role than Dub3 in stabilizing Snail protein (**Figure 2A**).

To determine the role of USP37 in maintaining Snail stability, we treated the 293T-Snail with CHX to block protein synthesis, and the protein level of Snail was examined using Western blotting assays. Consistent with prior reports, Snail was quickly degraded upon CHX treatment, while expression of USP37 markedly prevented Snail degradation (**Figure 2B**).

To determine if the enzymatic activity of USP37 is required to prevent Snail degradation, we made a mutant bearing the USP37. Cys350 residue to serine (USP37-C350S), which abolishes the enzymatic activity of USP37. Consistently, along with increasing amount of USP37 expression, the level of Snail protein was also increased, while USP37-C350S failed to prevent Snail protein degradation (**Figure 2C**).

To examine if USP37 affects the ubiquitination level of Snail, 293T-Snail cells transiently transfected with USP37 wild type (WT) or USP37-C350S or mock control were treated with MG132 to preserve the ubiquitinated substrates. The total Snail protein was immunoprecipitated with a specific antibody, and the ubiquitinated Snail was detected by using a specific antibody against ubiquitin. Notably, USP37 WT decreased the level of ubiquitinated Snail, while the USP37-C350S mutant showed no apparent effect (**Figure 2D**). Taken together, these data clearly demonstrate that USP37 stabilizes Snail protein *via* deubiquitination.

### USP37 Promotes Lung Cancer Cell Migration Through its Enhanced Enzymatic Activity

**Figure 3:**
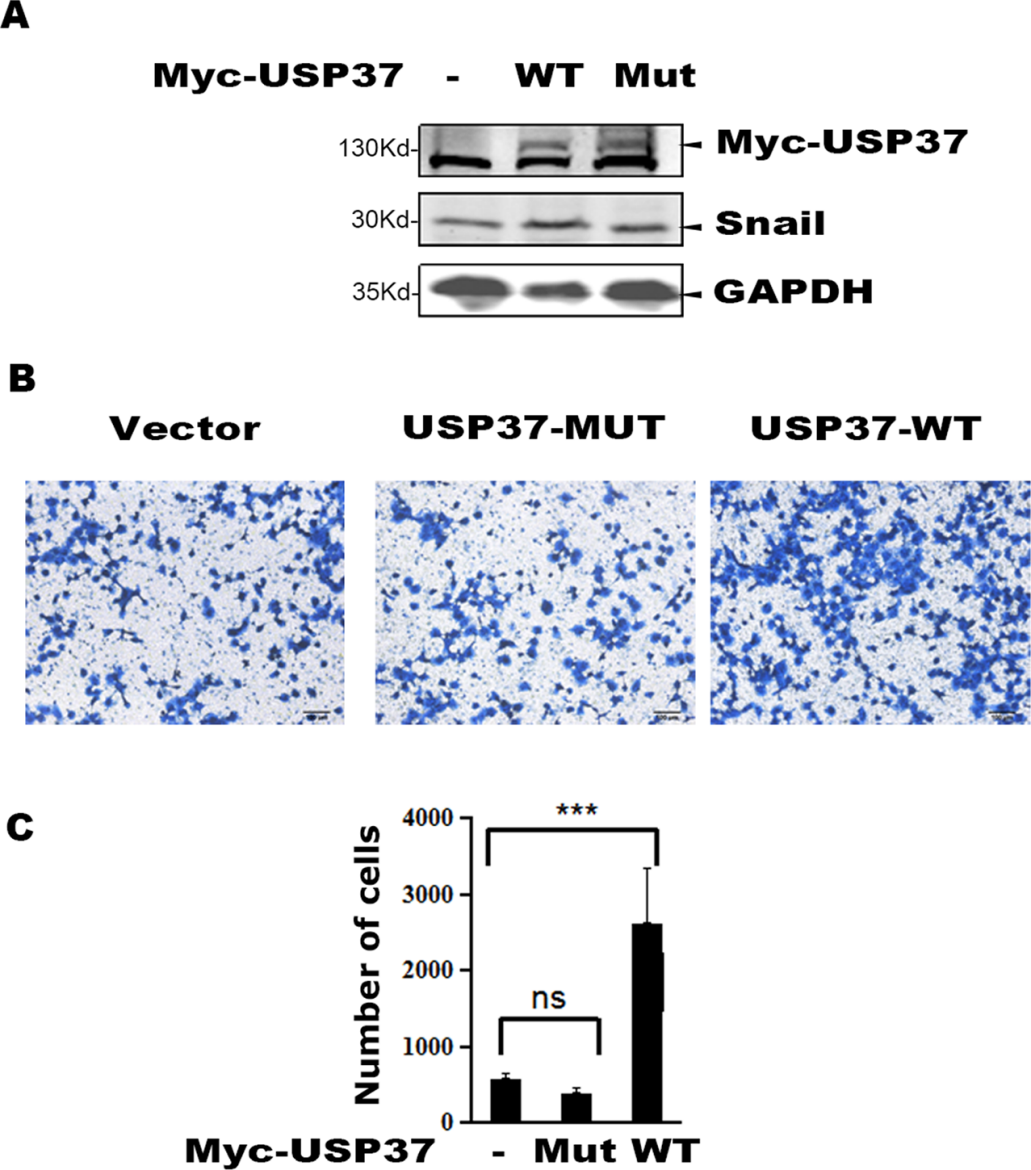
Overexpression of USP37 promotes cell H1299 cell migration. **(A)** H1299 cells were transfected with USP37 wild type (WT), USP37 mutant (MUT), and control vector. Cells were harvested 48 h post-transfection and whole cell extracts were prepared for Western blot assays to detect the Snail and USP37 protein levels. **(B)** Transwell assays show that USP37 can promote cell migration, while USP37 MUT fails to promote cell migration. Scale bars, 50 μm. **(C)** Quantitative statistics of the Transwell results of **(B)** ***P < 0.001 and NS, not significant.

To examine the role of USP37 in cell migration, we expressed USP37 WT and USP37-C350S mutant in lung cancer H1299 cells, and the transfected cells were prepared for Western blotting and cell migration assays. As reported, expression of USP37 WT increased the level of Snail protein (**Figure 3A**) and concomitantly stimulated cell migration (**Figures 3B, C**), while expression of USP37-C350S mutant showed no apparent effect on both Snail protein and cell migration (**Figure 3A**).

### Snail Is a Critical Substrate for USP37 to Enhance Cell Migration

**Figure 4:**
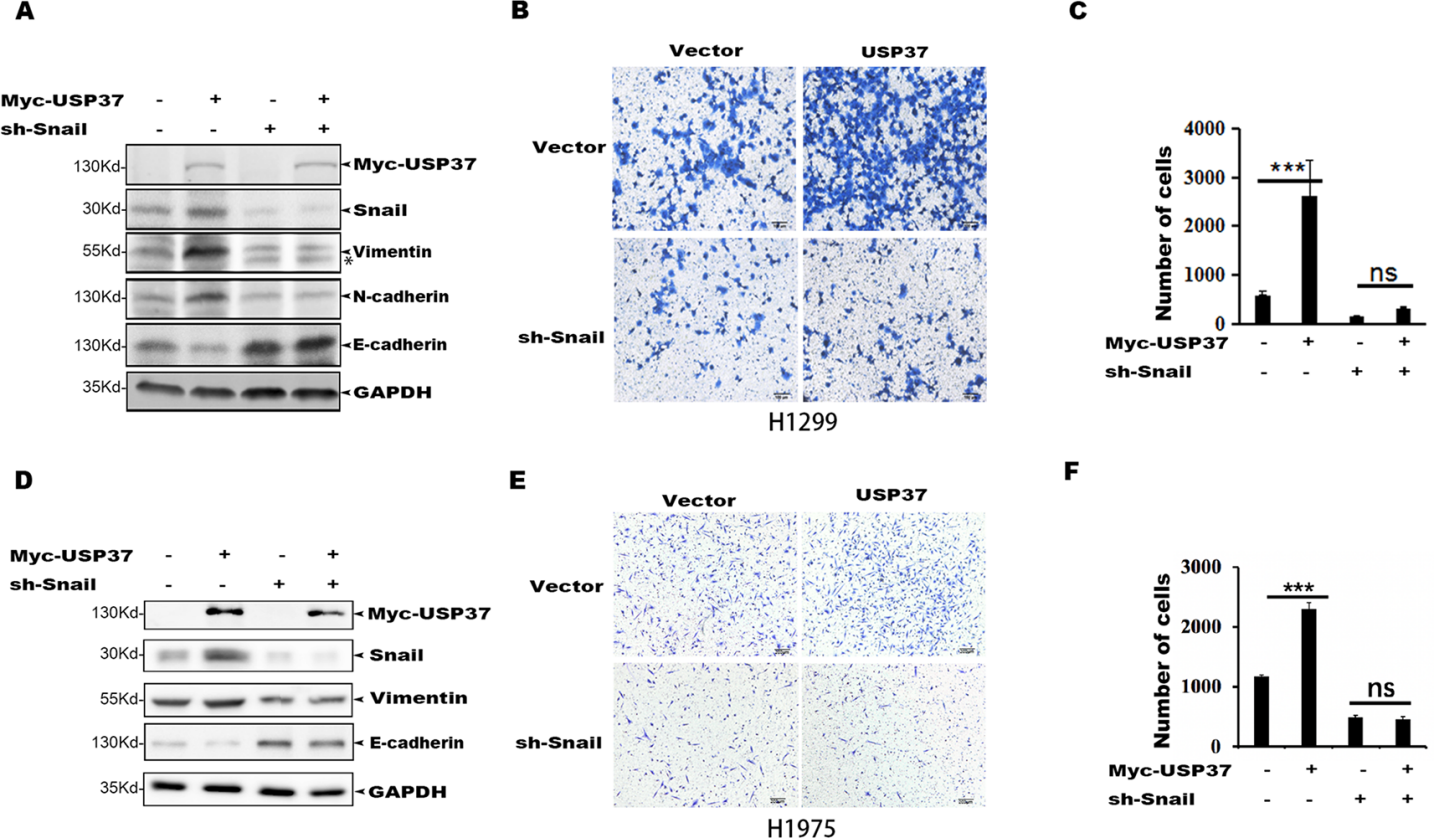
Depletion of Snail abolishes USP37-induced cell migration. **(A)** H1299 cells were first stably expressed short hairpin RNAs (shRNAs) specifically targeting Snail, and the resulting cells were then transfected with USP37 plasmids. The expressions of Snail, N-cadherin (N-cad), E-cadherin (E-cad), and vimentin (VIM) were examined by Western blot assays. *Non-specific band. **(B**, **C)** Transwell assays were performed simultaneously with ***P < 0.001 and NS, not significant. Scale bars, 50 μm. **(D)** H1975 cells were first stably expressed shRNAs specifically targeting Snail, and the resulting cells were then transfected with USP37 plasmids. The expressions of Snail, N-cad, E-cad, and VIM were examined by Western blot assays. **(E**, **F)** Transwell assays were performed simultaneously with ***P < 0.001 and NS, not significant. Scale bars, 50 μm.

To determine if Snail is an important substrate for USP37-mediated cell migration, we depleted Snail expression in H1299-USP37 and H1975-USP37 cells using an shRNA specifically targeting Snail. Western blotting assays showed that Snail protein was markedly decreased in the shRNA-transfected groups (**Figures 4A, D**). Notably, expression of USP37 resulted in elevated levels of vimentin and N-cadherin through stabilizing Snail, while depletion of Snail abolished the effect of USP37 on vimentin and N-cadherin (**Figures 4A, D**). Conversely, the expression of E-cadherin showed the opposite pattern to vimentin and N-cadherin (**Figures 4A, D**).

**Figure 5:**
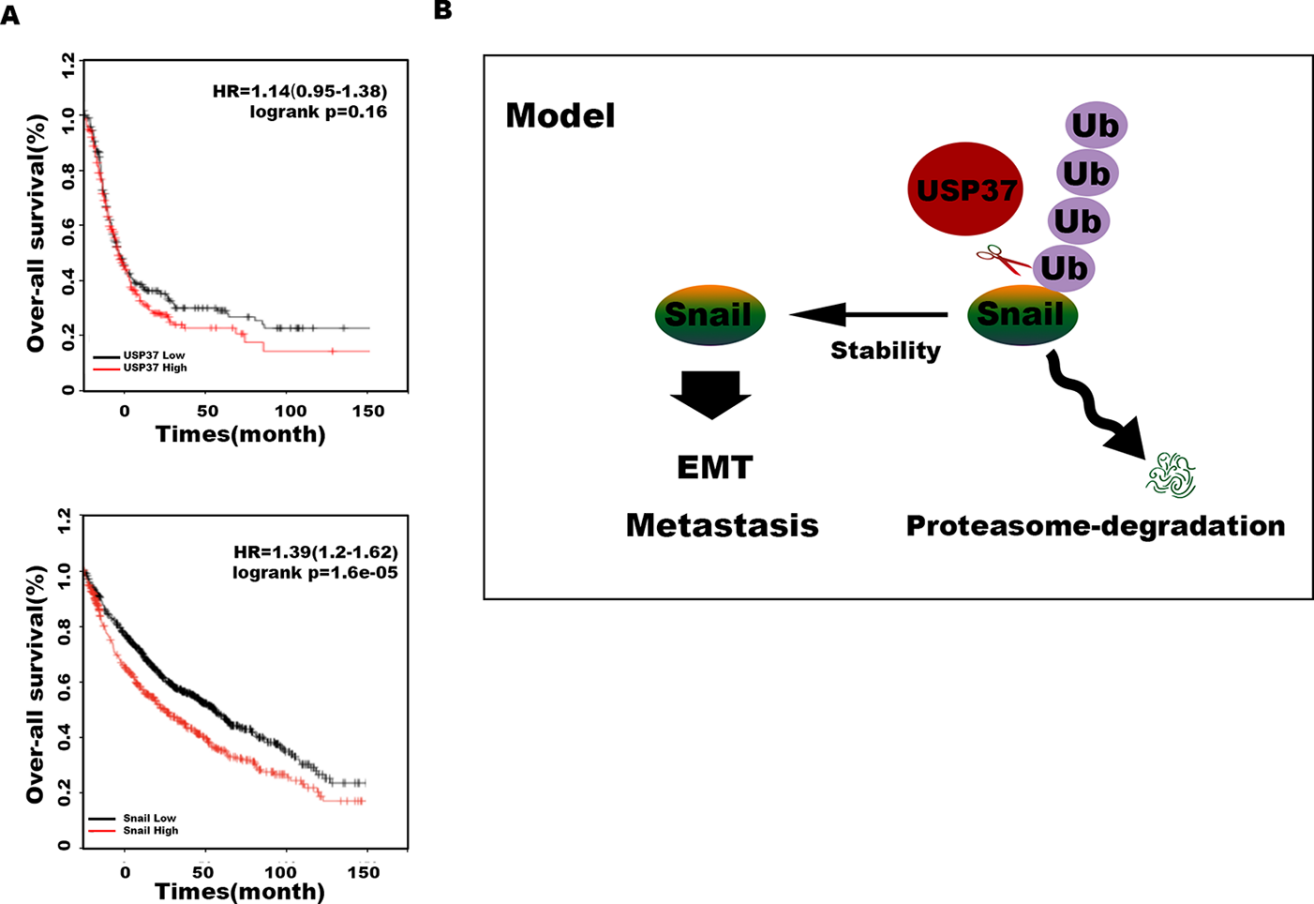
USP37 stabilizes Snail protein. **(A)** Overall survival analyses of all patients with lung cancer classified into high and low expression of USP37 (*up*) and Snail (*down*) were performed by Kaplan–Meier plots and log-rank tests. *Red*, high; *black*, low. **(B)** Diagram shows that USP37 promotes cell migration *via* preventing Snail degradation.

Transwell assays showed that depletion of Snail expression abolished the promotion effect of USP37 on cell migration (**Figures 4B, C, E, F**). In addition, high level of Snail and USP37 in non-small cell lung carcinoma (NSCLC) patients showed poor survival (**Figure 5A**). These data suggest that USP37 may be a new therapeutic target to treat lung cancer and prognosis marker for lung cancer patients. Collectively, these data indicate that USP37 promotes cell migration through stabilizing Snail *via* deubiquitination (**Figure 5B**).

## Discussion

Snail is a prominent EMT transcription factor and promotes EMT and tumor metastasis (Micalizzi et al., 2010; Diaz et al., 2014). Snail is upregulated in various types of cancer cells, and high level of Snail expression predicts poor survival and recurrence (Hwang et al., 2011; Hwang et al., 2014; Cercelaru et al., 2017; Cho et al., 2019; Wang et al., 2019). However, Snail protein is unstable and is quickly degraded through ubiquitination-mediated proteasome pathway predominately. Thus, DUBs are important for cancer cells to maintain high level of Snail protein by overriding the ubiquitination-mediated degradation process. There are approximately 100 putative DUBs in human genome, and it is believed that multiple DUBs may contribute to the stability of Snail protein in cells. Lately, DUB3, OTUB1, and USP27X have been identified as specific DUBs to stabilize Snail and play important roles in Snail-mediated tumor metastasis. In this study, we discover that USP37 directly binds Snail and markedly improves Snail protein stability through its deubiquitinase activity. Several E3 ligases, such as Fbxl5 (Vinas-Castells et al., 2014), Fbxl14 (Vinas-Castells et al., 2010), Fbxw1 (Diaz and de Herreros, 2016), and Fbxo11 (Jin et al., 2015), have been identified to induce Snail degradation *via* ubiquitination, but the specific ubiquitination site in Snail has not been defined. To distinguish the roles of DUB3, OTUB1, USP27X, and USP37 in Snail protein stabilization, it is necessary to identify the potential deubiquitination site, although our data showed that USP37 displays a more efficient activity to Snail stabilization than that of DUB3.

USP37 structurally constitutes an N-terminal PH domain, an internal linker, and a C-terminal catalytic domain (Yu, 2011; Kim et al., 2015; Manczyk et al., 2019). The catalytic DUB domain contains a unique insert of three ubiquitin-interacting motifs (UIMs) (Manczyk et al., 2019); however, the exact roles of the UIMs in these USP enzymes remain elusive. Recent data reported that mutations in UIM2 or UIM3 of USP37 result in reduced binding to ubiquitinated substrates (Manczyk et al., 2019).

Recent data have reported that the expression of USP37 is elevated in various types of cancers, including breast and lung cancers, and is strongly correlated with the increased mortality rate and metastasis (Pan et al., 2015; Qin et al., 2018). USP37 is found to participate in many biologic processes such as cell growth, mitosis, DNA replication, migration, and EMT, as well as stemness and chemoresistance. As such, a number of substrates have been identified for USP37 to commensurate its biological functions. For example, USP37 can interact with Hedgehog pathway components Smo and Gli-1 to stabilize these proteins and is essential for stemness maintenance, cell invasion, and EMT in breast cancer (Qin et al., 2018). In lung cancer cells, USP37 promotes cell proliferation and enhances the Warburg effect by directly interacting with c-Myc to deubiquitinate c-Myc (Pan et al., 2015). More importantly, high expression level of USP37 is associated with poor survival and prognosis of lung cancer patients, where the high expression level of Snail is also associated with poor survival and prognosis. We speculate that the expressions of USP37 and Snail in lung cancer are positively correlated. We will explore this hypothesis with NSCLC samples further. Additionally, USP37 also stabilizes CDK2, p27, CDT1, RAP80, and 14-3-3s to regulate cell cycle, DNA replication, and DNA repair, respectively (Huang et al., 2011; Kim et al., 2015; Typas et al., 2015; Das et al., 2016; Hernandez-Perez et al., 2016; Typas et al., 2016).

Our data showed that USP37 directly binds and deubiquitinates Snail and can markedly prevent Snail degradation; biologically, USP37 promotes lung cancer cell migration, and Snail is an essential substrate for USP37-mediated cell migration.

In summary, these data suggest that USP37 plays important roles in tumorigenesis and metastasis and may be a new therapeutic target to treat cancer and prognosis marker for NSCLC patients. However, how USP37 is upregulated in NSCLC and how these identified USP37 substrates cooperate to regulate cell growth and migration still need to be explored.

## Data Availability Statement

The raw data supporting the conclusions of this article will be made available by the authors, without undue reservation, to any qualified researcher.

## Author Contributions

Conception and design: HJ, ZH, and SL. Acquisition of data: JC and ML. Analysis and interpretation of data (e.g., statistical analysis, biostatistics, and computational analysis): JC, XW, LL, and QL. Writing, review, and/or revision of the manuscript: ZH, HJ, and SL. Administrative, technical, or material support: ML and JC. Study supervision: HJ, ZH, and SL.

## Conflict of Interest

The authors declare that the research was conducted in the absence of any commercial or financial relationships that could be construed as a potential conflict of interest.
